# Social contagion, the psychiatric symptom pool and non-suicidal self-injury

**DOI:** 10.1192/bjb.2024.101

**Published:** 2025-10

**Authors:** Joel Paris

**Affiliations:** 1 McGill University, Montreal, Canada; 2 Sir Mortimer B. Davis Jewish General Hospital, Montreal, Canada

**Keywords:** Suicide, self-harm, epidemiology, child and adolescent psychiatry, transcultural psychiatry

## Abstract

There is evidence that social contagion plays a role in shaping the clinical presentation of some psychiatric symptoms, particularly affecting features that vary over time and culture. Some symptoms can increase so rapidly in prevalence that they become ‘epidemic’. The mechanism involves a spread through peers and/or the media. Within broader domains of psychopathology, this process draws from a ‘symptom pool’ that can determine which specific symptoms will appear. This article illustrates these mechanisms by focusing on non-suicidal self-injury (NSSI), a syndrome that has been subject to social contagion and whose prevalence may have increased among adolescents.

Mental disorders and psychological symptoms can have rapid increases in prevalence over a very short time in both clinical and community populations, and this process is particularly common during adolescence.^1^ But rapid changes require an explanation. Since human genomics and neurobiology do not change over a few decades, we need to consider other possibilities.

This article will focus on mechanisms that reflect a process of social contagion.^2^ This construct describes how behaviours, emotions and other psychological phenomena spread spontaneously from one person to another within social groups. More simply, it refers to the tendency for people to mimic the behaviour of others in the same network.

Social contagion need not be the only mechanism leading to rapid changes in prevalence. It is also possible that, owing to stigma, some symptoms or behaviours remain hidden until they are too severe to ignore. However, this hypothesis does not account for the fact that the most severe mental disorders, such as psychoses, carry the highest level of stigma but are not associated with major changes in prevalence.^3^

Another possibility is that a change in exposure to risk factors, such as the frequency of psychosocial adversities, could account for increases in specific symptoms. For example, researchers have hypothesised that recent increases in depression and anxiety in adolescents could be due to the effects of social media.^4^ Although these risk factors tend to affect those who are already vulnerable, there is evidence that they can be pathogenic.

Still another explanation could be that changes in prevalence occur because the categories of disorder described in diagnostic manuals do not have sharp borders, tend to overlap and can easily shift over time. This process underlies the over-diagnosis of categories that are currently popular, both among physicians and patients. Thus, although changes in prevalence can reflect bottom-up processes, in which symptoms shift in the community owing to social influences, they can also reflect top-down processes in which clinicians define clinical phenomena in ways that lead to ‘diagnostic epidemics’.^5^ The availability of information on the web may encourage self-diagnosis, and some of these diagnostic fads also reflect patient preferences. There are other striking examples in which social contagion likely plays a role in diagnosis, such as eating disorders and chronic fatigue syndrome.

## Social contagion

Social contagion is not a completely new idea. One related concept is the phenomenon of ‘epidemic hysteria’, in which dramatic somatic symptoms begin in one individual, but are then replicated by others through a social network.^6^

Psychiatric symptoms can also be variable over time. Some clinical pictures are common at specific historical periods or in specific social settings, but are later replaced by other symptoms leading to different diagnoses. Thus, ‘hysteria’ was a common diagnosis in the 19th century, but can no longer be found in any diagnostic manual, and even at its height of influence, this syndrome presented at times with sensory changes, at other times with motor symptoms and at still other times as chronic fatigue.^7^ It has long been known that symptoms can vary greatly between cultures and that in more traditional societies psychological distress tends to be explained at a somatic level.

Adolescence is the developmental stage at which contagion is most prominent and peer influence becomes more powerful. For example, a recent large-scale study in a community population showed that friends of adolescents with mental disorders are much more likely to develop a diagnosis over time.^1^ Moreover, drawing on the symptom pool can be a way of making sense out of psychological distress.^8^ This mechanism has been further exaggerated by increasing use of social media: high levels of screen time are particularly common among those with mental health problems.^4^ The mechanisms behind social contagion include social learning theory, cognitive biases (such as bandwagon effects), social network dynamics, memetic transmission, echo chambers, as well as normative social influences and peer groups.

## Social contagion and the symptom pool

Shorter^7^ provided a strong theoretical framework that helps to account for the process of social contagion in psychiatry. His construct of a ‘symptom pool’ states that at any given historical point, societies tend to favour some symptoms over others as a way of expressing distress. These choices also need to be understood by describing multiple mechanisms of social contagion, with the crucial element deriving from peer influences. Thus, if a patient is in a social network with peers who use substances, who cut themselves, or starve themselves to be thin, they can be more likely to develop the same symptoms.^8^ Moreover, drawing on the symptom pool can be a way of making sense out of psychological distress. However, social contagion is most likely to affect people who are already vulnerable but who could have become symptomatic in a number of other ways.

Mental disorders of all kinds have often carried a degree of stigma, but some conditions have more stigma than others. Thus, over the course of history and across many societies, mental illness has often remained hidden, unless its effects on functioning become intolerable or life-threatening. If depression is experienced as fatigue, it is less likely to be seen as sign of personal weakness and serves to avoid stigma.

Another factor that can support social contagion derives from the fact that mental disorders lack biological markers and are more like syndromes than medical illnesses. Even so, patients can become strongly attached to what they may call ‘my diagnosis’, using a label to explain a wide variety of problems, sometimes to the point that a diagnosis can become part of their identity. In this way, social contagion can be reinforced by health professionals who prefer certain diagnoses and who encourage patients to frame their problems to support these preconceptions.

## Non-suicidal self-injury (NSSI)

A systematic review^9^ has documented an important relationship between social contagion and non-suicidal self-injury (NSSI) that suggests how social influences are shaped by a psychiatric symptom pool. NSSI usually begins in early adolescence, shortly after puberty; it is not suicidal in intent but is used to suppress or control negative emotions. Long-term follow-up studies show that most adolescents with NSSI give up self-harm over time, so that these behaviours can be described as intermittent and experimental.^10^ But those who continue to self-harm can go on to develop features of adult mental disorders, particularly if they also have high levels of emotion dysregulation at baseline.

A large-sale epidemiological study of adolescents in the USA^11^ reported a 12-month community prevalence of 7.3% for NSSI in adolescents, and data have been quite similar in other countries. Although these numbers seem to point to an increase in NSSI in adolescents, in the absence of longitudinal data, it is not possible to document how much of an increase we are seeing.

Thus, NSSI is a prime example of social contagion. The internet has been a powerful medium for social contagion of psychological symptoms, and it may have increased the overall level of distress among adolescents.^4^ Those with NSSI may find validation from online friends who have the same behaviour and/or from websites that promote it. Adolescents would be less likely on their own to come up with such a way to reduce emotion dysregulation.

The vehicles for social contagion are multiple, but one mechanism probably derives from the direct influence of peers. However, until there is more systematic research, the jury is out as to whether social contagion is the main factor driving increased prevalence of NSSI or a trigger that opens the door to this syndrome.

## Summary

When clinical symptoms or mental disorders rapidly increase in prevalence, social contagion should be considered as a likely mechanism shaping changes in the form of psychopathology. This seems to be part of the process that drives the emergence of NSSI in adolescents or young adults. But what look like increases in prevalence may also reflect forces influencing choices from the symptom pool.

## About the author

Joel Paris, MD, is Emeritus Professor in the Department of Psychiatry, McGill University, Montreal, and a Research Associate in the Department of Psychiatry, Sir Mortimer B. Davis Jewish General Hospital, Montreal, Quebec, Canada.

## Data Availability

Data availability is not applicable to this article as no new data were created or analysed in this study.

## References

[1] Alho J, Gutvilig M, Niemi R, Komulainen K, Böckerman P, Webb RT, et al. Transmission of mental disorders in adolescent peer networks. JAMA Psychiatry 2024; 81: 882–8. 10.1001/jamapsychiatry.2024.1126 PMC11112494 38776092

[2] Sooknanan J, Comissiong DM. When behaviour turns contagious: the use of deterministic epidemiological models in modeling social contagion phenomena. Int J Dyn Control 2017; 5: 1046–50.

[3] Kirkbride JB, Croudace T, Brewin J, Donoghue K, Mason P, Glazebrook C, et al. Is the incidence of psychotic disorder in decline? Epidemiological evidence from two decades of research. Int J Epidemiol 2009; 38: 1255–64. 18725359 10.1093/ije/dyn168 PMC3307031

[4] Twenge JM. Does online social media lead to social connection or social disconnection? J College Character 2013; 14: 11–20.

[5] Frances A. Saving Normal: An Insider's Revolt against Out-of-Control Psychiatric Diagnosis, DSM-5, Big Pharma, and the Medicalization of Ordinary Life. William Morrow, 2013.

[6] Zhao G, Cheng Q, Dong X, Xie L. Mass hysteria attack rates in children and adolescents: a meta-analysis. J Int Med Res 2021; 49(12): 3000605211039812. 10.1177/03000605211039812 PMC8829737 34898296

[7] Shorter E. From Paralysis to Fatigue: A History of Psychosomatic Illness in the Modern Era. Free Press, 1992.

[8] Dishion T, Tipsord J. Peer contagion in child and adolescent social and emotional development. Ann Rev Psychol 2009; 62: 189–214. 10.1146/annurev.psych.093008.100412 PMC3523739 19575606

[9] Jarvi S, Jackson B, Swenson L, Crawford H. The impact of social contagion on non-suicidal self-injury: a review of the literature. Arch Suicide Res 2013; 17: 1–19. 23387399 10.1080/13811118.2013.748404

[10] Moran P, Coffey C, Romaniuk H, Olsson C, Borschmann R, Carlin JB, et al. The natural history of self-harm from adolescence to young adulthood: a population-based cohort study. Lancet 2012; 379: 236–43. 22100201 10.1016/S0140-6736(11)61141-0

[11] Taliaferro LA, Muehlenkamp JJ, Borowsky IW, McMorris BJ, Kugler KC. Factors distinguishing youth who report self-injurious behavior: a population-based sample. Acad Pediatr 2012; 12: 205–13. 22424698 10.1016/j.acap.2012.01.008

